# Novel intraoperative near-infrared imaging strategy to identify abnormalities in the anterior mediastinum

**DOI:** 10.1186/s13019-022-02054-8

**Published:** 2022-12-09

**Authors:** Sonia Singhal, Feredun Azari, Gabriel C. Caponetti, Gregory T. Kennedy

**Affiliations:** 1 Department of Surgery, University of Pennsylvania Perelman School of Medicine, Pennsylvania, PA USA; 2 Department of Pathology and Laboratory Medicine, University of Pennsylvania Perelman School of Medicine, Pennsylvania, PA USA; 3 Department of Surgery, Hospital of the University of Pennsylvania, 3400 Spruce Street, Philadelphia, PA 19104 USA

**Keywords:** Mediastinum, Lymph node, Staging, Intraoperative molecular imaging

## Abstract

Thoracic surgeons are frequently asked to biopsy suspicious tissues in the anterior mediastinum to discriminate between a reactive versus malignant pathology such as lymph nodes. The most common benign cause of a mediastinal lymph node is a reactive lymph node from a prior infection or inflammatory process such as post-COVID or granulomatous disease. The most common malignant cause is a lymphoproliferative disorder but also metastatic disease from neck, breast and other regional cancers. Biopsies in this location are challenging because they are far from the trachea and the sternum is a barrier to most diagnostic procedures. Thus, a surgical biopsy is frequently required and a common procedure for Thoracic surgeons. Technically, identifying these lesions can be challenging, particularly for small lesions or those in patients with high body mass index. In order to improve contrast between diseased tissue in the anterior mediastinum and surrounding adipose tissue, we have been studying near-infrared imaging during surgery using indocyanine green (ICG) to give contrast to the abnormal tissues and to avoid an unnecessary extended resection. We developed a modified technique to give ICG to a patient during a biopsy in the anterior mediastinum to specifically highlight abnormal tissues. As a proof-of-principle, we present a case of a young woman with a suspicious 2 cm mediastinal lymph node that required surgical biopsy.

## Introduction

Thoracic surgeons are frequently consulted to diagnose abnormalities in the anterior mediastinum such as enlarged mediastinal lymph nodes, metastases, thymic lesions, ectopic thyroid lesions and germ cell tumors. The most common abnormalities in the anterior mediastinum are enlarged lymph nodes due to reactive or malignant processes. Enlarged reactive lymph nodes can develop due to infections from the head and neck (e.g. Ludwig’s angina) as well as the lung (e.g. post-COVID infection), non-specific hyperplasia, infectious and non-infectious granulomatous disease (e.g. tuberculosis, sarcoidosis) and inflammatory processes (e.g. lupus) [1, 2]. Mediastinal hematolymphoid neoplasms are the most common malignant cause of anterior mediastinal masses [3]. These typically include classic Hodgkin lymphoma, primary mediastinal large B-cell lymphoma, mediastinal gray zone lymphoma, diffuse large B-cell lymphoma, marginal zone lymphoma, anaplastic large cell lymphoma, T-lymphoblastic lymphoma/leukemia, and plasmacytoma. The other common possibility is metastatic cancer that has tracked to the anterior mediastinum such as a prior lung cancer or breast cancer in inner quadrants of the breast.

Obtaining access to the anterior mediastinum to biopsy these abnormalities is a clinical challenge. Bronchoscopy is typically utilized for the middle mediastinum but cannot reach the anterior mediastinum. Transthoracic approaches are restricted by the sternum and mammary vessels. Another limitation of both these needle based approaches is the lack of sufficient tissue to obtain complete morphologic assessment. Furthermore, anterior mediastinal nodules are typically encased in adipose tissue and can be difficulty to tease out from the thymus and pericardial fat [4].

Our group has been studying an adjunct technology to visually enhance nodules called fluorescence guided surgery (FGS) [5]. FGS is under development for multiple cancers including non-small cell lung cancer [5], head and neck cancer, ovarian cancer and brain tumors [6]. However, there is no prior reports of utilizing this technology for abnormalities in the anterior mediastinum which is a major clinical challenge. FGS is the process of injecting an optical contrast agent into the patient prior to surgery that localizes to nodules. Then, during the surgery, a camera capable of detecting fluorescence is used to identify the optical signal from the nodule and discriminate it from surrounding normal tissue. Over the last decade, several fluorescent conjugates have emerged that target specific receptors on cancer cells [7–9]. However, this approach is not helpful for identifying tissues such as lymph nodes that are either reactive or have cancer cells that do not express a particular receptor. Thus, a novel method to identify abnormalities in the mediastinum is lacking at this time.

Our proposed novel approach requires a fluorescent dye that does not target a specific receptor, but instead, localizes to abnormal tissues, regardless of whether they are cancerous or not. In order to accomplish this, our group has been exploring features common to abnormal or inflammatory tissues. An important commonality is the increased vascular permeability that occurs in inflammatory lesions. The release of bradykinin, wide fenestrations in the endothelial cells, altered interstitial pressure and electrical polarity characterize inflammatory tissues, irrespective of their malignant or benign pathology. Prior work in targeting nanoparticles to these inflammatory tissues using these properties has been termed the enhanced permeability and retention (EPR) effect [10, 11].

Based on preclinical studies on the EPR effect, we hypothesized that a fluorescent nanoparticle, indocyanine green, would be the optimal agent to identify inflamed nodules. We have found that indocyanine green (ICG) when given at high doses should visually enhance mediastinal lymph nodes [10, 12]. In this report, we present a clinical case of a 2 cm suspected mediastinal lymph node that was avid on PET scanning in the anterior mediastinum in a patient with a body-mass-index of 31 kg/m^2^. Given the clinical challenges of this case, we opted to utilize FGS with ICG to localize the lymph node and obtain a rapid diagnosis.

## Case report

The patient was 56-year-old female with a body-mass-index of 31 kg/m^2^ who presented one year ago with classic Hodgkin lymphoma, nodular sclerosis subtype, diagnosed on a left supraclavicular lymph node biopsy. She was ultimately diagnosed as Stage IIB and underwent chemotherapy with doxorubicin (Adriamycin), bleomycin, vinblastine, and dacarbazine (ABVD). She tolerated the treatment well and was felt to be in remission. Six months later, she was diagnosed with a coronavirus SARS-CoV2. This required hospitalization but ultimately she was discharged with minimal pulmonary sequelae.

**Fig. 1:**
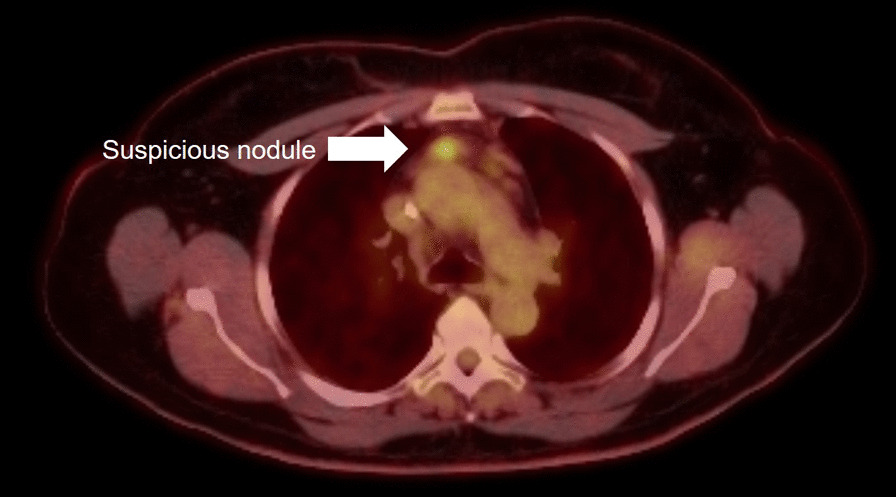
PET imaging of the anterior mediastinum identified a 2.2 cm prevascular lymph node with an SUV of 4.7

**Fig. 2:**
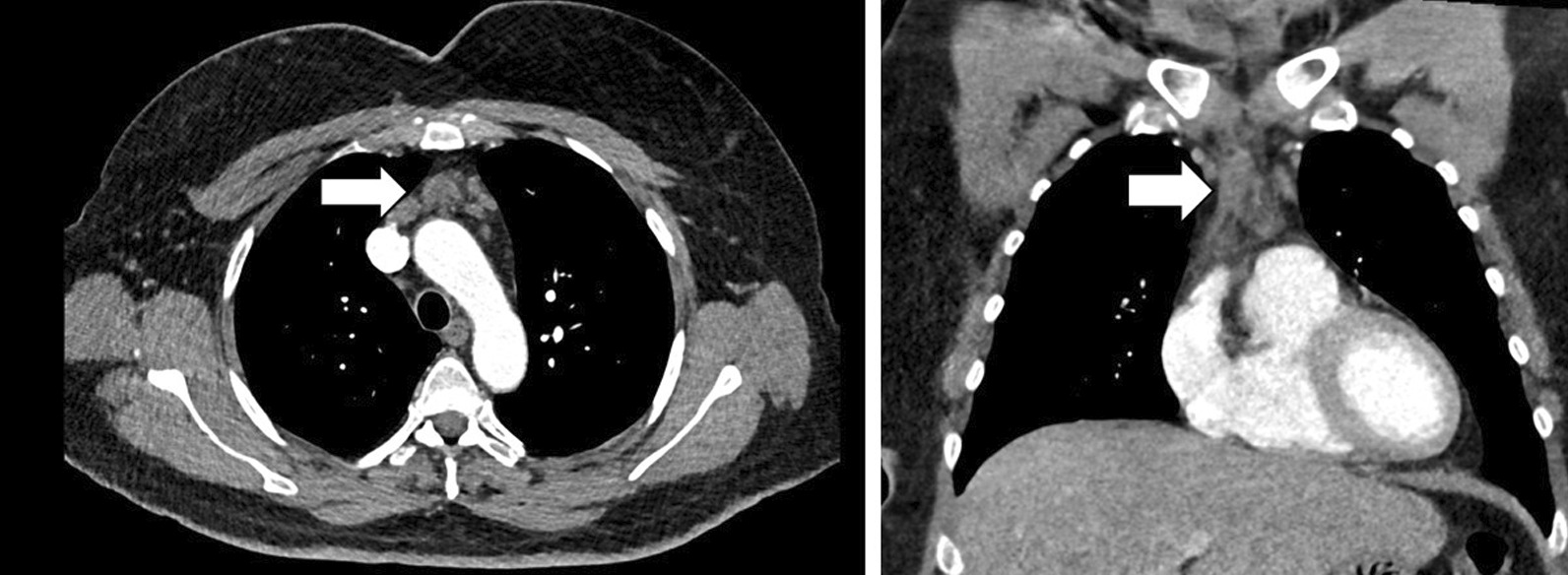
Contrast enhanced CT of the chest was performed using thin section reconstructions. The patient was given intravenous contrast with iopamidol. A 1.8 cm prevascular lymph node was seen on axial and sagittal views

Six weeks after discharge, she underwent surveillance chest and abdominal positron emission tomography (PET) scanning and was discovered to have a 2.2 cm anterior mediastinal nodule with a standardized uptake value (SUV) of 4.7 (Fig. 1). On the PET scan, no other lesions were evident. The nodule was in the prevascular space and was not amenable to a needle biopsy. A three-week interval computed axial tomograph (CAT) scan confirmed the lesion and was measured at 1.8 cm in size (Fig. 2). At presentation, the differential included a reactive lymphadenopathy due to recent SARS-CoV2 infection versus recurrent lymphoma. The patient was referred for consultation with a thoracic surgeon for surgical biopsy to guide therapy.

On presentation, the patient was asymptomatic. Her relevant medical history included a history of tuberculosis status post isoniazid treatment. Her prior medications included bleomycin, though she had no evidence of pulmonary disease. She was a lifelong non-smoker and had no second hand smoking exposures. On physical exam, her lung was clear to auscultation. No other lymph nodes were palpable in her supraclavicular fossa, axillae or groins.

A decision was made to undergo a right robotic approach to the mediastinum. Due to the small size of the lesion and body mass index of 31 kg/m^2^, she was counseled that there would be a high risk of inability to locate the nodule. In order to improve the likelihood of visualizing the nodule, she was informed that she would be injected with 25 mg of indocyanine green (ICG) intravenously thirty minutes prior to the case.

**Fig. 3:**
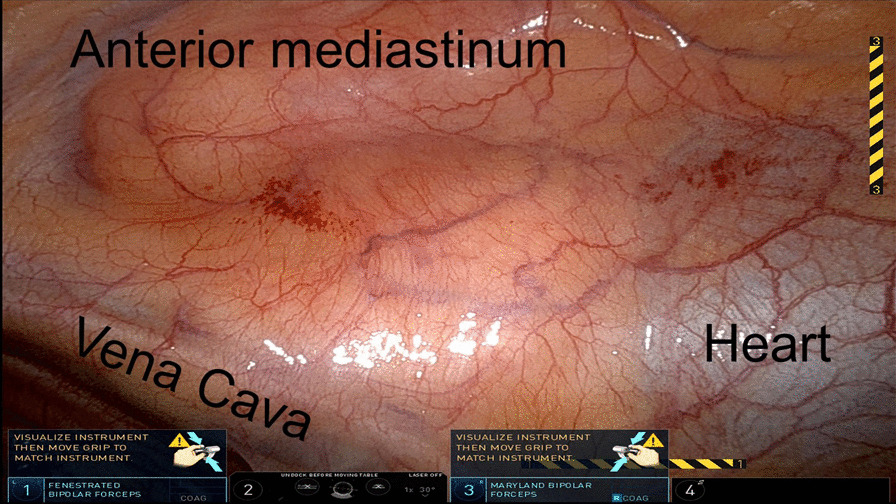
White light imaging of the mediastinum showed no obvious sign of the mediastinal lesion. The anterior mediastinum, vena cava and heart are seen from the perspective of the right hemithorax

**Fig. 4:**
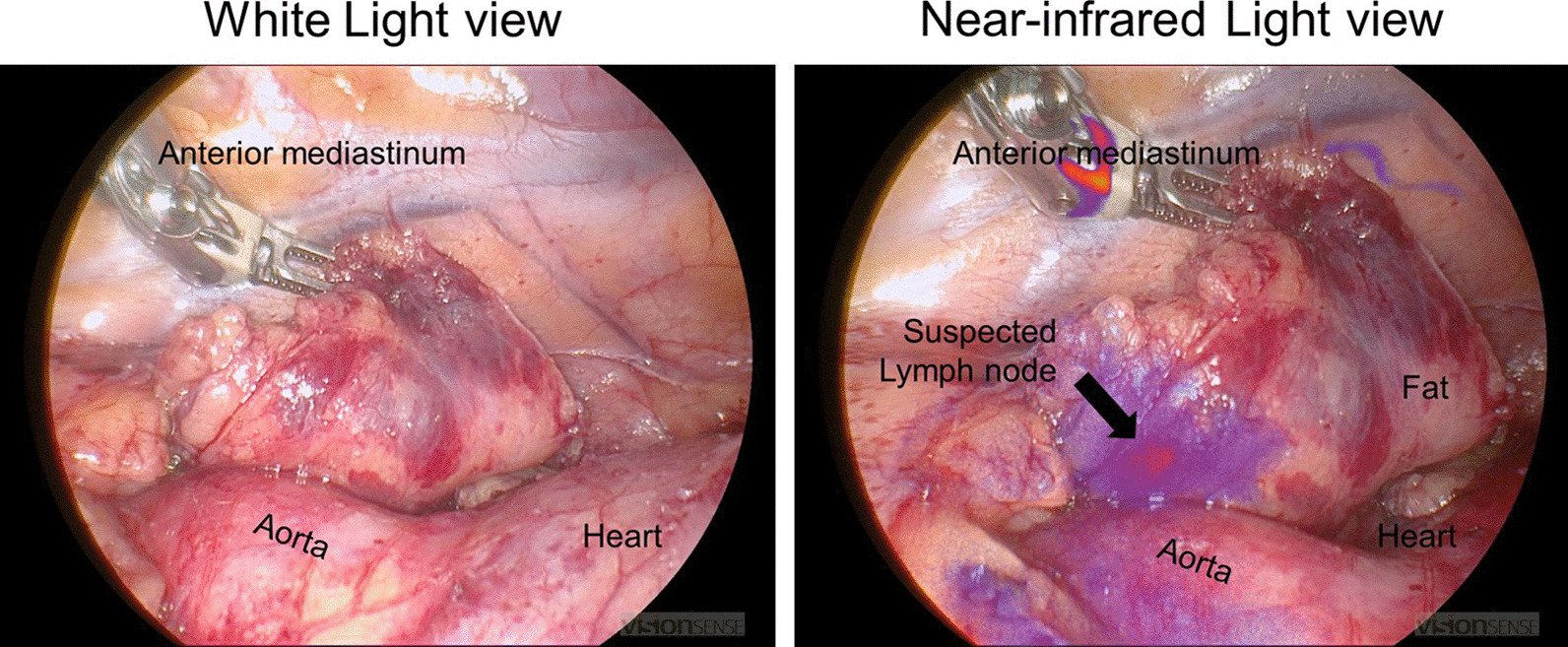
White light imaging did not reveal the location of the diseased tissue in the anterior mediastinum. However, near-infrared imaging localized the abnormal tissue distinctly for the surgeon to do a targeted resection

**Fig. 5:**
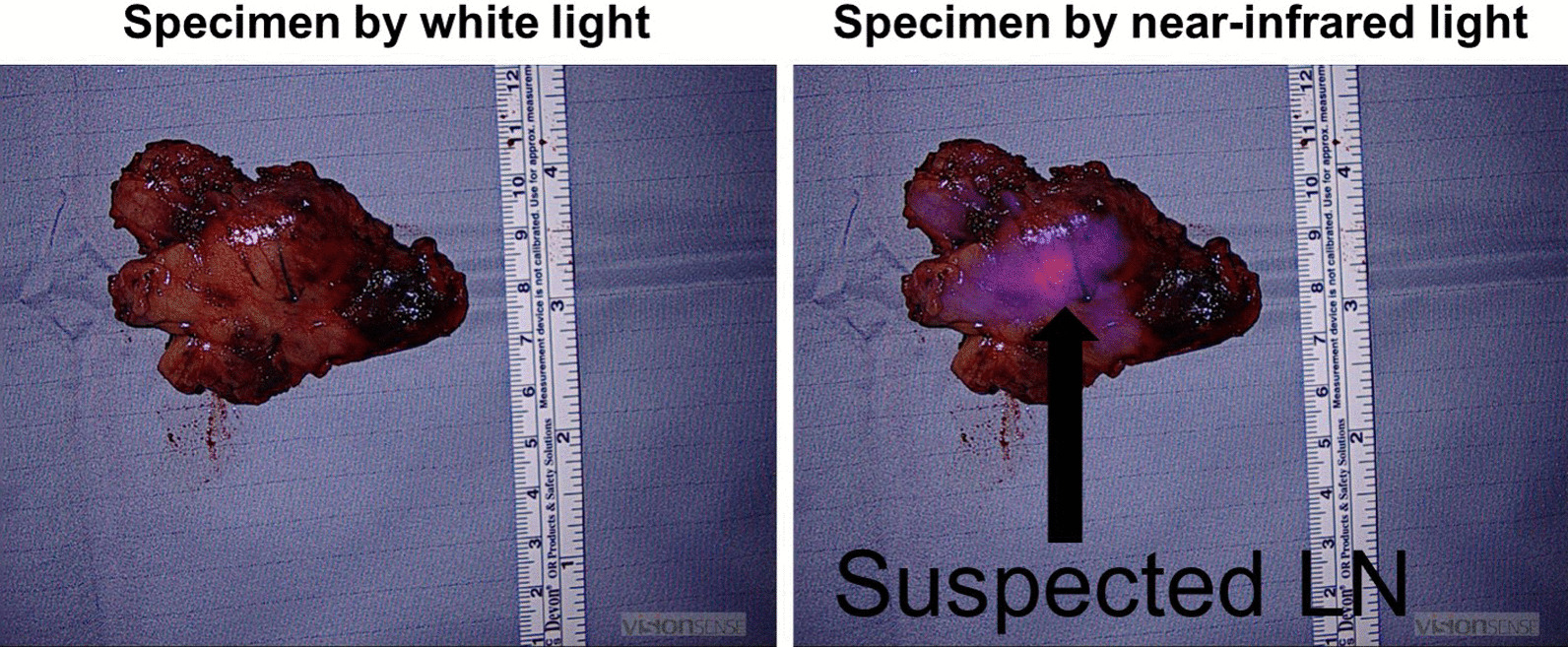
On the back table, the fluorescence from the abnormal tissue which was a suspected lymph node contrasted well from the surrounding fatty tissues. The ratio of fluorescence from the lesion to the fat was 6.3

During the operation, the nodule could not be visualized by the standard white light DaVinci endoscope (Fig. 3). The endoscope was then toggled to a near-infrared mode that allows highlighting of any lesions that fluoresce over 800 nm (“Firefly mode”). Upon toggling to the Firefly mode, the nodule in the mediastinum was visualized. A Medtronic Visionsense Iridium system was also tested to identify the lesion (Fig. 4). The lesion and surrounding tissue was excised without difficulty without extending the magnitude of the operation because of the localization by FGS. After excision, the nodule was visualized with a near-infrared microscope and fluorescence as identified in the lesion but not the normal surrounding fat (Fig. 5). The ratio of fluorescence from the lesion to the fat was 6.3 (ImageJ, NIH).

**Fig. 6:**
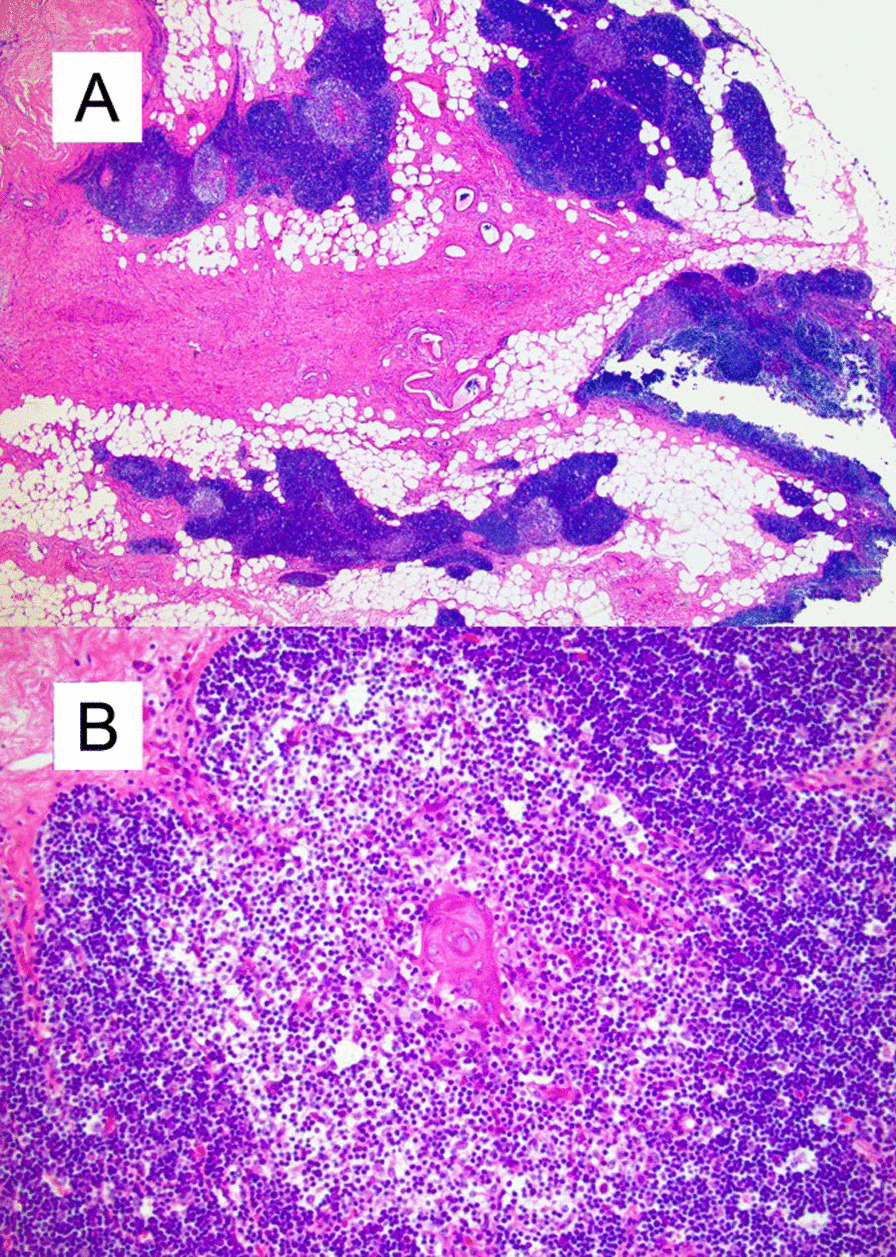
Histopathologic evaluation of the 2.2 cm anterior mediastinal nodule revealed diagnostic features of benign thymic hyperplasia with no evidence of recurrent lymphoma. **A** H&E-stained tissue sections show fragments of thymic tissue and fibroadipose tissue (H&E, 2.5 ×). **B** At higher magnification, the thymic tissue shows normal cortical and medullary architecture with occasional Hassall's corpuscles. Atypical cells (including Hodgkin/Reed-Sternberg cells) are not observed (H&E, 20 ×)

The patient achieved full post-surgical recovery and was discharged home the following day. The evaluation by pathology of the nodule revealed benign thymic hyperplasia without evidence of recurrent lymphoma (Fig. 6).

## Discussion

Challenges in thoracic operations include (i) identification of small primary, synchronous, or metachronous pulmonary nodules and cancers, and (ii) accurate selection of lymph nodes with abnormal pathology. Thoracic surgeons only have two intraoperative tools, visual inspection and manual palpation, to accomplish these tasks [5]. Fluorescence guided surgery (FGS) is an emerging technology with the potential to dramatically improve these operations. This technology requires (i) a fluorescent dye that will selectively accumulate in abnormal tissues, and (ii) a specialized imaging system to detect and quantify the fluorescence from the dye in the tissues.

Several features of FGS make it more attractive than other intraoperative adjuncts to a surgeon’s hands, eyes, and clinical decision making. First, it does not involve exposure to ionizing radiation. Second, the imaging is easy to interpret for surgeons unfamiliar with the technology. Third, it causes minimal interruption and integrates nicely into the normal flow of an operation.

Lymph nodes are a particularly challenging situation. Lymph nodes can be normal versus enlarged. Lymph node enlargement can be due to reactive or malignant processes. Enlarged lymph nodes share common features, regardless if they are reactive versus cancerous such as increased vascular permeability. The release of bradykinin, wide fenestrations in the endothelial cells, altered interstitial pressure and electrical polarity characterize inflammatory tissues, irrespective of their malignant or benign pathology. Prior work in targeting nanoparticles to these inflammatory tissues using these properties has been termed the enhanced permeability and retention (EPR) effect [10, 11].

The mechanism of indocyanine green (ICG) remains elusive. The EPR effect is the concept that nanoparticles cannot escape the tight junctions of normal capillaries but extravasate from the leaky capillaries of tumors [13, 14]. Due to properties such as size, shape, charge, and polarity, these molecules are then retained within the inflamed tissues [15]. Although ICG is a small molecule (0.775 kDa), it is 98% protein bound in circulation and therefore once it is protein bound, acts as a nanoparticle though the size of the endothelial gaps for EPR allow most particles below 200 microns [16, 17]. The ICG binds to tissues with increased angiogenesis, allowing both malignant and benign nodules to fluoresce [20]. ICG has excitation and emission wavelengths of approximately 805 nm and 830 nm, and it is the only clinically approved near-infrared fluorophore [11]. ICG has traditionally been used for vascular perfusion imaging; common applications include assessment of perfusion to tissue flaps and bowel anastomoses [18, 19]. When used to assess perfusion, a low dose of ICG (5–10 mg) is given as a bolus immediately prior to imaging.

More recently, investigators have demonstrated that NIR imaging with ICG can also detect hepatocellular carcinoma (HCC), colorectal and pancreatic cancer liver metastases, and sentinel lymph nodes in breast cancer and melanoma [20–24]. Currently, in hepatic tumors, it has been postulated that anion-transporting polypeptides, intracellular transporters and export transporters expressed on hepatocytes have been responsible for tumor contrast. In non-hepatic tumors, it is hypothesized that the EPR effect is the primary mechanism by which solid cancer accumulate ICG. The mechanism of EPR is related to differences in tumor oncotic pressure, pH, disorganization of vascular endothelium, local prostaglandin and bradykinin levels, and lack of lymphatic angiogenesis. [25, 26]

The clearance of ICG is a topic of major interest. Historically, ICG was considered to have a short half-life of 12 min. However, clinical data has shown otherwise. For example, when used for liver tumors, a low to medium ICG dose ranging from 10 mg to 0.5 mg/kg is given 1–7 days before surgery. ICG, which is cleared hepatically, then accumulates within the tumors due to impaired biliary excretion. Even at one week, the signal is still visible [10].

When used for sentinel lymph nodes, albumin-bound ICG (ICG:HSA) is given in peritumoral injections, and NIR imaging can highlight draining lymphatic channels and nodes. We postulated that given ICG’s properties, it could be given systemically because it should diffuse into any abnormal tissue that has developed an inflammatory response.

Thus, based on preclinical studies, for this patient we hypothesized that a high dose such as 25 mg could be given prior to surgery to achieve sharp contrast between abnormal mediastinal lymph nodes and the surrounding fat [12, 27]. We optimized this dose in preclinical models. We found that excessively high doses caused background from the retention of the ICG in the vasculature, specifically the aorta and heart. At lower doses, the fluorochrome washed out prior to visualization. This report was our first attempt to visualize a mediastinal lymph node using this dose and timing.

This case was an excellent demonstration of this technology for multiple reasons. First, this patient already had lymphoma so there was a high pre-test probability by the oncology team that this was recurrent lymphoma. However, prior to proceeding with empiric therapy which was considered, a decision was made to obtain tissue confirmation. This was ultimately the correct decision because the biopsy showed reactive thymic tissue. Second, it seemed clinically surprising that the patient would only have one reactive lymph node on the PET scan given the recent COVID infection. During multidisciplinary discussion leading up the biopsy, this was felt to be unlikely. Third, one of the controversies in FGS is uptake of contrast agents after chemotherapy because of the changes in the vascular permeability. Ultimately, this was not prohibitive so the case informed future research.

Ultimately, the lymph node was not cancer, and, in fact, the culprit was abnormal reactive thymic tissue. The fact that ICG injected in this fashion could identify reactive tissue provides important information that this technique may provide a solution to identifying both benign and reactive lymph nodes or abnormal tissues.

For future work, we will accumulate more cases to determine the safety, feasibility and efficacy of this approach. We will also conduct future research to determine a way to use ICG to identify malignant nodules within mediastinal masses. Another option in the future will be targeted dyes such as the recently FDA approved agent pafalocianine (Cytalux) [28]. This current agent is indicated for ovarian cancer because of the presence of folate receptor alpha on these cancer cells. However, an off-label use may be identifying tumor associated macrophages because they express folate receptor beta and would also endocytose this new contrast agent. Although this work has not been done, it might be feasible moving forward to consider these targeted or even activatable probes for these applications. If successful, this approach of FGS with ICG will have significant improvement to surgical biopsy of anterior mediastinal masses.

## Data Availability

All images available.
